# Up-conversion emission in transition metal and lanthanide co-doped systems: dimer sensitization revisited

**DOI:** 10.1038/s41598-023-28583-3

**Published:** 2023-02-07

**Authors:** Daniel Avram, Claudiu Colbea, Andrei A. Patrascu, Marian Cosmin Istrate, Valentin Teodorescu, Carmen Tiseanu

**Affiliations:** 1 National Institute for Laser, Plasma and Radiation Physics, PO Box MG-36, 76900 Bucharest-Magurele, Romania; 2 Scientific Center for Optical and Electron Microscopy, ETH Zürich, Zürich, Switzerland; 3 National Institute of Materials Physics, 405A Atomistilor Street, 077125 Magurele-Ilfov, Romania; 4 Faculty of Physics, University of Bucharest, 077125 Magurele, Romania; 5 Academy of Romanian Scientists, 050094 Bucharest, Romania

**Keywords:** Nonlinear optics, Nanoparticles, Optical spectroscopy, Nanoparticles

## Abstract

Lanthanide (Ln) co-doped transition metal (TM) upconversion (UC) co-doped systems are being intensively investigated for their exciting applications in photonics, bioimaging, and luminescence thermometry. The presence of TM, such as Mo6 + /W6 +, Mn2 +, or Fe3 + determines significant changes in Ln UC emission, such as intensity enhancement, colour modulation, and even the alteration of the photon order. The current mechanism assumes a ground-state absorption/excited-state absorption (ESA/GSA) in TM-Yb dimer followed by direct energy transfer to Er/Tm excited states. We revisit this mechanism by addressing two issues that remain ignored: a dynamical approach to the investigation of the upconversion mechanism and the intrinsic chemical complexity of co-doped TM, Ln systems. To this aim, we employ a pulsed, excitation variable laser across a complete set of UC measurements, such as the emission and excitation spectra and emission decays and analyze multiple grains with transmission electron microscopy (TEM). In the Mo co-doped garnet, the results sustain the co-existence of Mo-free garnet and Mo oxide impurity. In this Mo oxide, the Er upconversion emission properties are fully explained by a relatively efficient sequential Yb to Er upconversion process, with no contribution from Yb-Mo dimer sensitization.

## Introduction

Co-doping lanthanide (Ln) based nanoparticles with transition metals (TM) is intensively exploited in the search for upconversion (UC) systems with high quantum efficiency and modulated colour^1–8^. TM, especially Mo(6 +), but also W(6 +) (both considered as d^0^ centred anion Mo/WO_4_^2−^), d^5^ Mn(2 +) and Fe(3 +) determine significant intensity enhancement paired with colour change and photon order modification of Ln UC emission in many hosts such as Yb_2_Ti_2_O_7_, YbAG^9^, TiO_2_ and Gd_2_O_3_, Bi_7_Ti_4_NbO_21_^10^, Al_2_O_3_^11,12^, Y_2_Mo_4_O_15_^13^, ZnO^14^, NaY(Lu)F_4_^15,16^ and KZnF_3_^17^. As such, Mo co-doping enhances the Er UC emission by two orders of magnitude coupled with a colour change from red (Mo free) to green^9^. Also, Mn(2 +) enhances the Er green UC emission by two orders of magnitude in Mn, Er-YbAG and by a factor of 30 in Mn, Er/Tb, Yb-NaY(Lu)F_4_^15,16^. Finally, Fe(3 +) enhances the Er red UC emission by 7 in Fe, Yb, Er-NaYF_4_^18^.

Unlike co-doping with alkaline metal (especially Li^5,19^), which enhances the Ln UC emission without modifying the UC mechanism, e.g. Yb to Er/Tm energy transfer, TM co-doping changes the nature of the UC mechanism. The current mechanism assumes a ground state absorption followed by excited-state absorption (GSA/ESA) in the TM-Yb pair followed by direct energy transfer to Er/Tm excited levels. Since GSA/ESA is a one-ion process, an exchange-coupled dimer model is used where neighbouring, exchange-coupled Yb-TM ions represent the chromophore unit^20,21^. This interpretation represents an adaptation of the previously well-documented UC mechanism occurring in Yb-Mn dimer UC in halide hosts^22,23^. In this case, theoretical and experimental works demonstrated the occurrence of the exchange interaction between pairs of Yb and Mn bridged by halide ligands in restrictive geometry^24^.

Because of the ability to control both the intensity and colour of Ln UC, TM co-doping is praised as a solid strategy for optimizing UC emission with foreseen applications in displays, lasers, photonics^17^, bioimaging^16,25^, solar cells^26^ thermometry^9,27–29^, pH sensor^15^. Despite the increased interest in TM, Ln UC systems, a detailed understanding of the underlying mechanism, which is mandatory for designing new materials, is not available. In this work, we revisit the current interpretation by considering two neglected issues using a different experimental approach to that employed so far. We mention that the UC emission in mixed TM, Ln systems (recently reviewed in^30^) exceeds the scope of this paper, which is focused exclusively on the Ln UC emission explained by the TM-Yb sensitization process.

The first issue relates to the efficiency of the GSA/ESA UC, which is known to be up to three orders of magnitude^9^ weaker than the ETU-based UC. This makes the reported significant enhancement of Ln emission by Yb-TM dimer sensitization questionable. Further, given the high sensitivity of the exchange interaction not only to Yb-TM distances but also to the geometry of the Yb-ligand (here, oxygen/fluoride based)-TM^31^ arrangement, it is unlikely that such a variety of host structures enable efficient Yb-TM exchange interactions. Even for the halide compounds for which the Yb-Mn dimer upconversion emission is well-demonstrated, the efficiency drops by orders of magnitude from 28 to 0.05% when the Yb-Mn bridging geometry changes from purely corner-sharing bridging to face-sharing connectivity^31^.

The second issue is intrinsic to heterovalent doping with low solubility metals (Mo, Mn)^32^. The valency and ionic radii mismatch can prevent the complete incorporation of TM on the lattice sites. The low solubility of Mn(2 +) in the rare-earth oxides/fluorides leads to vast differences (up to two orders of magnitude) between the nominal and measured concentrations^32^. On the other side, the Mo–Ln–O systems have complex chemistry, which determines various structural types depending on the Mo/Ln ratio, temperature, and the specific rare earth cation involved^33^. With few exceptions and only by increasing the Mo concentration above the value currently in studies mentioned above^34^, X-ray diffraction data do not reveal the presence of a TM-related phase impurity. Besides, the formation of a homogenous solid solution where Mo, Yb, and Er elements distribute uniformly on the nanoscale range was not demonstrated so far by high-resolution transmission electron microscopy (TEM) investigations on multiple grains.

Unlike fixed wavelength cw excitation, pulsed excitation can easily discriminate between GSA/ESA and ETU mechanisms^35^ by comparing the Ln emission decay under upconversion (Yb absorption) and direct excitation (Ln activator absorption). Such an approach is definitely more appropriate when investigating the potential multisite, dopant, or phase segregation effects induced by TM, which are known to impact the Ln luminescence intensity, colour, and dynamics^36^.

As a case example, we selected the system previously investigated in Ref.^9^. We employed a pulsed and tunable laser spanning the complete Yb absorption and relevant Er absorption levels. The Er upconversion emission and excitation spectra and emission decays were measured under identical energy density, enabling a correct comparison between Mo-free and Mo co-doped systems. In addition, local structure investigations using Eu as a luminescence probe were also pursued. High-resolution TEM on multiple grains were analyzed in detail. The results evidence a molybdenum oxide containing Yb and Er elements, which is notably absent in standard X-ray diffraction and optical absorption being associated with a local Mo enrichment by TEM analysis on multiple grains. Our work dismisses the current interpretation grounded in the Yb-Mo dimer, illustrating the critical role of the experimental approach in the correct interpretation of the UC mechanisms in chemically complex systems.

## Materials and methods

### Materials

**Er/Eu**_**0.25**_**Yb**_**2.75**_**Al**_**5**_**O**_**12**_ and **Er/Eu**_**0.25**_**Mo**_**1**_**Yb**_**1.75**_**Al**_**5**_**O**_**12**_ samples were prepared by a sol–gel method^1^ followed by annealing at 1250 °C. Firstly, Al(OC_3_H_7_)_3_ was dissolved in a ratio of 1:2 in an equimolar mixture of acetylacetonate and isopropyl alcohol. A mixture of water and isopropyl alcohol (water to Al molar ratio = 0.85:1) is added to the formed gel, and HNO_3_ is used to adjust the pH to 3 in order to prepare the solution for RE precursor (Er/Eu(NO_3_)_3_ × 5H_2_O; Yb(NO_3_)_3_ × 5H_2_O; (NH_4_)_6_Mo_7_O_24_) addition. The resulted solution is dried at 100 °C under vacuum and annealed for 1 h at 1250 °C with a heating ramp of 2 °C/min. **Yb**_**2**_**(MoO**_**4**_**)**_**3**_ doped with 1%Eu samples were synthesized by a solid-state reaction employed on another report^2^. The Yb_2_(MoO_4_)_3_: 1%Eu phosphor was prepared using 113 mg MoO_3_, 102 mg Yb_2_O_3_, 2.4 mg Eu(NO_3_)_2_‧5H_2_O and 11 mg NH_4_F‧HF (considering 5% wt. flux agent). After mixing all the reagents together, they were calcinated at 800 °C for 4 h.

### Characterization

Powder X-ray diffraction (XRD) patterns were recorded on a Shimadzu XRD-7000 diffractometer using Cu Kα radiation (λ = 1.5418 Å, 40 kV, 40 mA) at a scanning speed of 0.10°/min in the 10–90 2θ range. The crystallite size was estimated using the Scherrer equation. Diffuse reflectance DR-UV–Vis spectra were recorded at room temperature on an Analytik Jena Specord 250 spectrophotometer with an integrating sphere for reflectance measurements and MgO as the reflectance standard. DR–UV–Vis spectra of the samples were recorded in reflectance units and were transformed in Kubelka–Munk remission function F(R). TEM characterization was done using an analytical transmission electron microscopy JEM ARM 200F and JEM-F200-F2 at an acceleration voltage of 200 kV. The TEM specimen preparations were performed by sample powder mechanical grinding and deposition on holey carbon copper grids.

### Luminescence measurements

*In steady-state excitation measurements*, a continuous-wave (cw) *up-conversion emission*, a 973 nm fibre-coupled diode laser system (RLTMFC-980-4W-5, ROITHNER LASERTECHNIK GmbH) with a bandwidth of ~ 5 nm was used. The digital photographs were taken with a Canon EOS 60D camera with an exposure time of 2.5 s and 1600 ISO. A FESH 750 (short pass filter) was used to monitor only the UC signal.

*In pulsed excitation measurements*, a wavelength tunable NT340 Series EKSPLA OPO (Optical Parametric Oscillator) for samples excitation at 210 ÷ 2300 nm with 10 Hz pulse repetition. Laser spectral width was around 5 cm^−1^ with a pulse duration of < 5 ns. As a detection system, an intensified CCD (iCCD) camera (Andor Technology, iStar iCCD DH720) coupled to a spectrograph (Shamrock 303i, Andor) with 0.33 nm spectral resolution was used. The digital photographs were taken with a Canon EOS 60D camera with 1/2.5 s exposure time and 1600 ISO.

*The upconversion excitation spectra* were obtained by monitoring the Er green and red emissions with 1 ms gate width and 0.1 µs delay after the laser pulse under scanning the Yb absorption between 875 and 1050 nm with 1 nm step^3,4^.

*The down-conversion excitation spectra* measurements were carried out using a Fluoromax 4/Fluorolog 3 spectrofluorometer (Horiba) operated in fluorescence mode. The monochromator slits were constant in the excitation mode (1 nm) and varied from 0.2 to 1 nm in the emission mode.

*The emission decays* were measured using a PMT module (PMA-C 192-N-M, PicoQuant GmbH) coupled to a monochromator (Shamrock 505i, Andor) and a PCIe TCSPC card TimeHarp 260 NANO (PicoQuant GmbH) as an acquisition system. The average decay times were estimated by integrating the area under the normalized emission decays.

## Results and discussion

### Overview of structure and UC emission properties of (Mo), Er-YbAG under cw excitation

**Figure 1:**
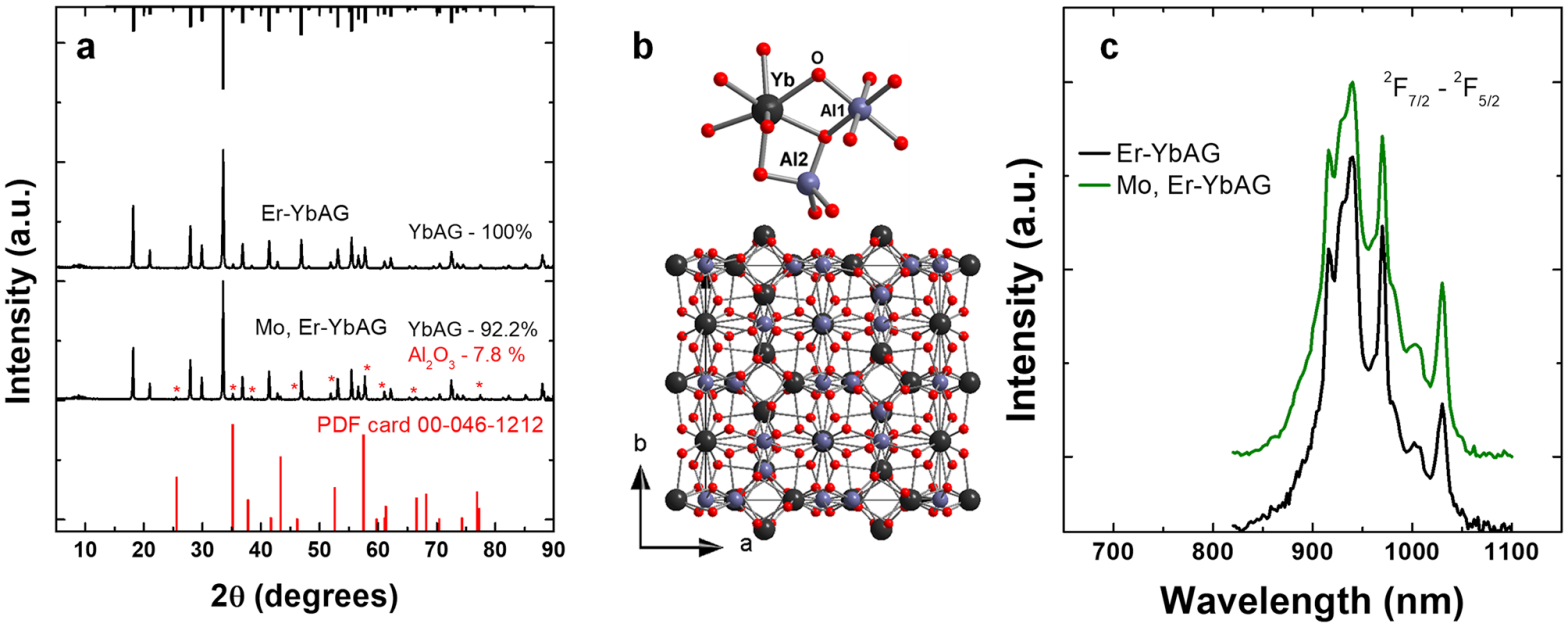
(**a**) XRD patterns, (**b**) Structural representation of YbAG lattice and (**c**) Diffuse reflectance spectra of (Mo) Er- YbAG in the region of Yb ^2^F_7/2_-^2^F_5/2_ absorption transition. All XRD patterns and Diffuse reflectance spectra were normalized at maximum intensity for comparison purposes.

The Er_0.25_Yb_2.75_Al_5_O_12_ (8.33% at. Er and 91.67% at Yb) and Er_0.25_Mo_1_Yb_1.75_Al_5_O_12_ (12.5% at. Er, 87.5% at. Yb), denoted as Er-YbAG and Mo, Er-YbAG were prepared by a sol–gel method^9^ described in Supplementary Information. The X-ray diffraction patterns of Mo-free and Mo co-doped Er YbAG (Fig. 1a) are consistent with a cubic cell of YbAG structure with a space group of $Ia\overline{3}d$ (JCPDS No. 23-1476).

Mo increases the lattice constant slightly from 11.936 to 11.939 Å and the crystallite size (estimated by Scherrer Equation^37^) from about 47 to 55 nm. The error in the lattice constant calculations is generally below 0.01%. The stoichiometry for Er_0.25_Mo_1_Yb_1.75_Al_5_O_12_ sample changes with the ratio of Er to Yb increasing from 8.33 to 12.5% at. leading to a slight increase of the lattice constant^38^.

Weak diffraction peaks indexed as Al_2_O_3_ observed in (Mo), Er-YbAG contribute up to 7.8% (estimation made using MAUD software, Supplemental Information). Generally, rare-earth garnets can retain the garnet crystalline structure with some deficit of Al_2_O_3_^39^, especially in the case of Lu and Yb^40^ garnets. Within the garnet lattice, Yb and Al cations occupy dodecahedral 24(c) sites and tetrahedral 24(d) octahedral 16(a) sites, respectively, and the oxygen atoms occupy the regular 96(h) positions (Fig. 1b), leading to the formula of Yb_3_Al_2_Al_3_O_12_, commonly written as Yb_3_Al_5_O_12_. Although garnets possess a cubic crystal structure, they offer a complex arrangement of different cations in the unit cell tolerant of doping with both lanthanide and metal ions with 2 + , 3 + and 4 + valences^41–43^. Thus, the dodecahedral site can be occupied by trivalent rare-earth and divalent Ca, while the octahedral site can be occupied by trivalent or divalent (Mg and Mn). Finally, along with trivalent Ga or Al cations, the tetrahedral site can tolerate divalent Mn and tetravalent metals such as Si, and Ge, with many other substitutions, also possible^44^. The ionic radii of Al and Yb are about 0.51 and 0.86 Å. The ionic radius of Mo^6+^ is 0.62 Å, which is thus closer to that of Al. However, it is unlikely that Mo^6+^ cations replace the trivalent Al with the subsequent formation of an Al vacancy for charge neutrality. Mo^6+^ cations substitute more likely the Yb cations in the dodecahedral site with two Yb ions replaced with simultaneous formation of cationic voids in the dodecahedral sites. The diffuse reflectance spectra (Fig. 1c) show similar absorptions of (Mo), Er- YbAG with a maximum at 968 nm, which is close to the 0-phonon absorption line of ^2^F_7/2_ → ^2^F_5/2_ transition of Yb in YAG^45^. Mo does not alter the 800 to 1100 nm absorption profile, nor does it induce any additional absorption from 300 to 800 nm.

**Figure 2:**
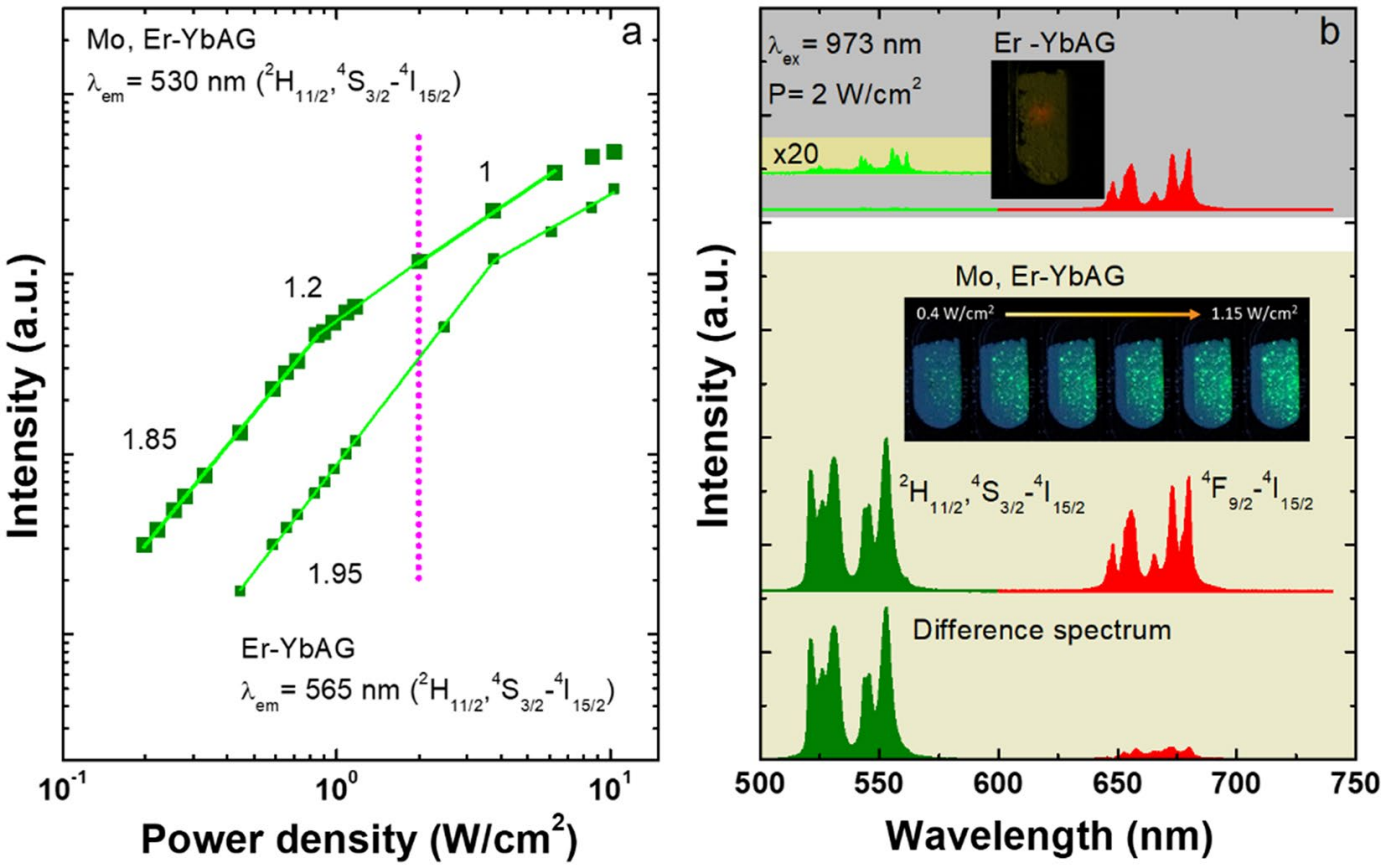
(**a**) Power density dependence of Er upconversion emission in (Mo) Er-YbAG under 973 nm cw excitation. Vertical dotted line corresponds to power density used in experiments (2 W/cm^2^); (**b**) Comparison between the Er upconversion emission spectra in (Mo)Er-YbAG. Digital photos show a non-uniform distribution of green (Er) bright spots across powder samples contained in a quartz cuvette.

Figure 2 gathers the power density dependencies and the UC emission spectra of Er-YbAG and Mo, Er-YbAG under 973 nm cw excitation.

For comparison purposes, the used power density was fixed at 2 W/cm^2^ (Fig. 2a) being limited by the relatively weak Er green emission (^2^H_11/2_, ^4^S_3/2_-^4^I_15/2_ transition) in Mo-free Er-YbAG. As estimated from the power density dependency law, ${I}_{UPC}\sim {P}^{n}$^46^, the photon order of green emission is close to 2 in Er-YbAG, which is reduced to 1.2 in Mo, Er-YbAG at the power density used in the experiments.

Without Mo, the Er UC emission is almost purely red with an intensity (I) ratio of red and green emission, R/G = I(^4^F_9/2_-^4^I_15/2_)/(^2^H_11/2_, ^4^S_3/2_-^4^I_15/2_) of 61. Mo co-doping shifts the emission colour from red to green with an associated R/G of 0.6 and intensifies the green emission roughly by two orders of magnitude (Fig. 2b), in good agreement with the trend reported in the literature^9,12,34,47^. We note that the emission enhancement induced by Mo is underestimated since the used power density of 2 W/cm^2^ falls within the semi-saturated regime of the more emissive Mo, Er-YbAG (Fig. 2a).

### UC emission properties of (Mo), Er-YbAG under variable pulsed excitation across Yb absorption.

It is apparent from Fig. 2 that besides colour and intensity changes of the UC emission, Mo broadens the Er emission, alters the Stark splitting pattern, and the relative emission intensity corresponding to the thermalized ^2^H_11/2_ and ^4^S_3/2_-^4^I_15/2_ transitions (Er). Such modifications, together with the naked-eye observation of bright green spots (see the digital photo in Fig. 2b), suggest that Mo determines a spatially non-uniform Er distribution in YbAG. To verify this assumption, we measured the Er UC emission spectra, energy density dependencies, and emission decays in Mo, Er-YbAG using a pulsed and tunable laser (laser beam profile illustrated in Fig. S1).

**Figure 3:**
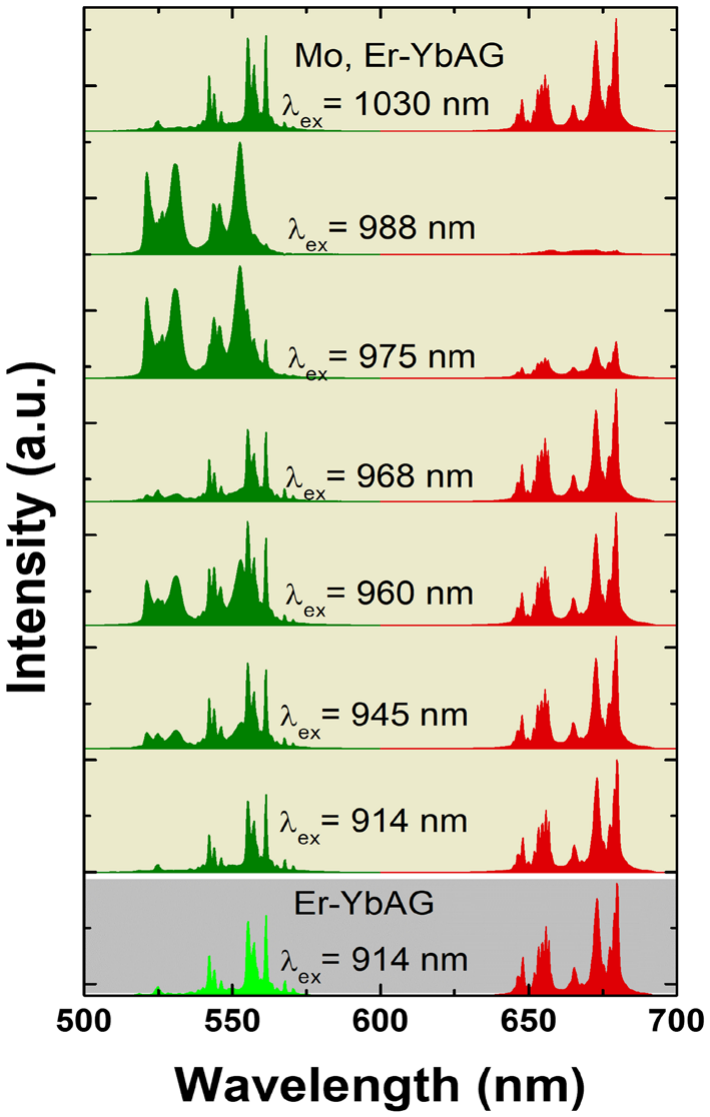
Upconversion emission spectra of Er-YbAG and Mo, Er-YbAG using variable pulse excitation (at 20 mJ/cm^2^) across Yb absorption (914–1030 nm).

**Figure 4:**
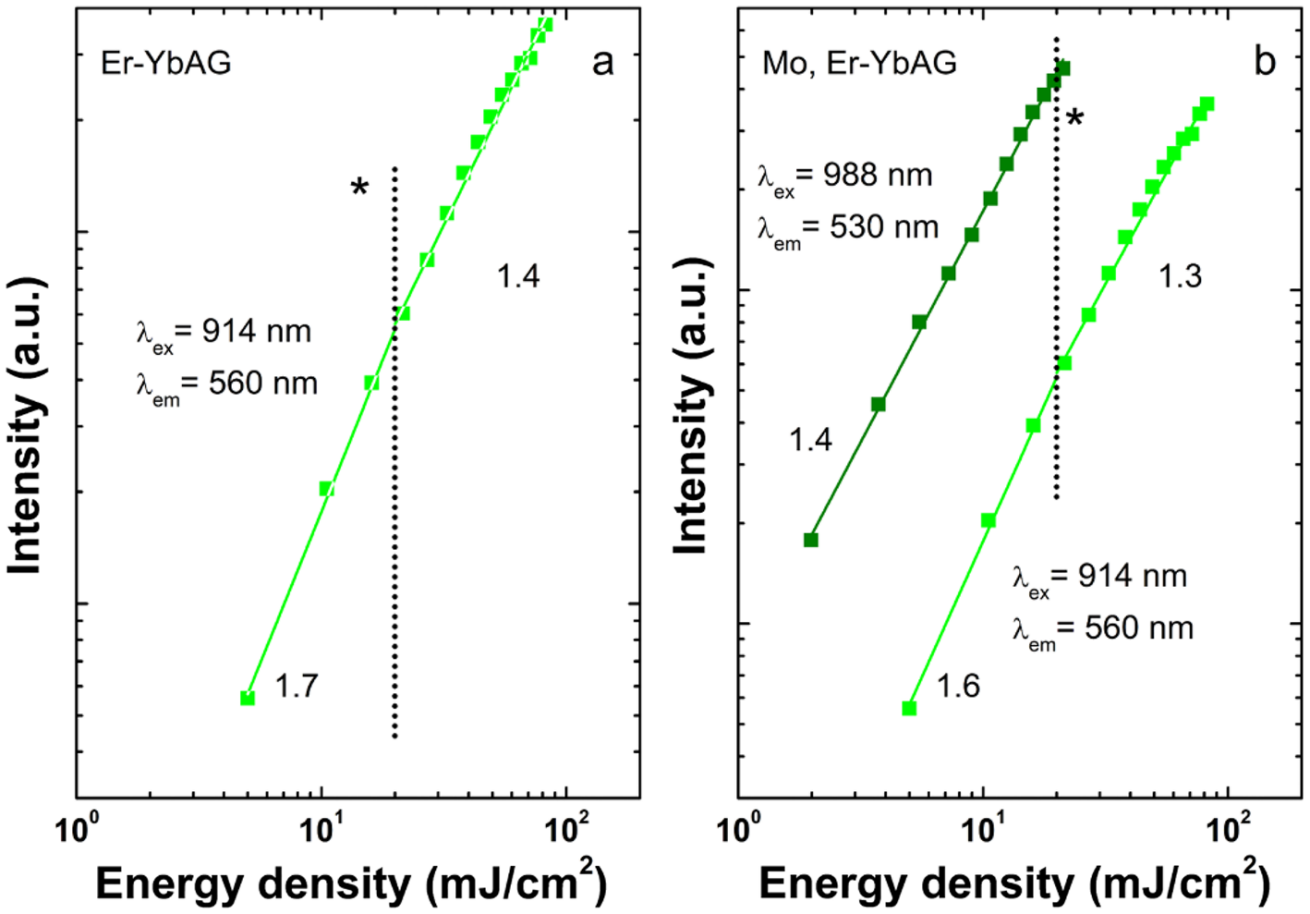
Energy density dependencies of Er green upconversion emission in Er-YbAG (**a**) and Mo, Er-YbAG (**b**) were monitored at 560 and 530 nm under 914 and 988 nm excitation, respectively. The vertical dotted line denoted the energy density of 20 mJ/cm^2^ used in the experiments.

Figure 3 presents a selection of the UC spectra measured using variable excitation across the Yb (^2^F_7/2_-^2^F_5/2_) absorption. The Yb absorption was laser scanned from 875 to 1050 nm with a 1 nm step at an energy density of 20 mJ/cm^2^, which, unlike the case of cw excitation, corresponds to the non-saturated regime of both Mo-free and Mo, Er-YbAG (Fig. 4).

Without Mo, the Er UC emission remains constant in shape (e.g. R/G ratio at 1.4), irrespective of the excitation wavelength. This result confirms a homogeneous distribution of Er onto YbAG D_2d_ lattice sites. It should be noted that, due to the short pulse duration of the excitation laser and the longer timescale of red emission compared to green emission (Fig. S2), Er-YbAG presents a smaller R/G ratio under pulsed excitation (1.4) compared to cw excitation (61). The highest UC emission intensity is reached for excitation at 968 nm, which matches the maximum Yb absorption (Fig. 1c).

By contrast, Mo addition significantly changes the shape of Er up-conversion emission when varying the excitation wavelength across Yb absorption. Spectral deconvolution indicates that the UC spectra consist of linear superposition of two spectra: one spectrum is identical to that measured with a Mo-free sample (best discriminated at 914 nm, R/G = 1.4); the second is almost purely green (R/G = 0.006) and is best discriminated at 988 nm. The UC emission shape measured under pulsed 988 nm excitation parallels that observed under cw excitation, showing relative broader emission with an intensity distributed roughly similar between the ^2^H_11/2_ and ^4^S_3/2_-^4^I_15/2_ transitions.

**Figure 5:**
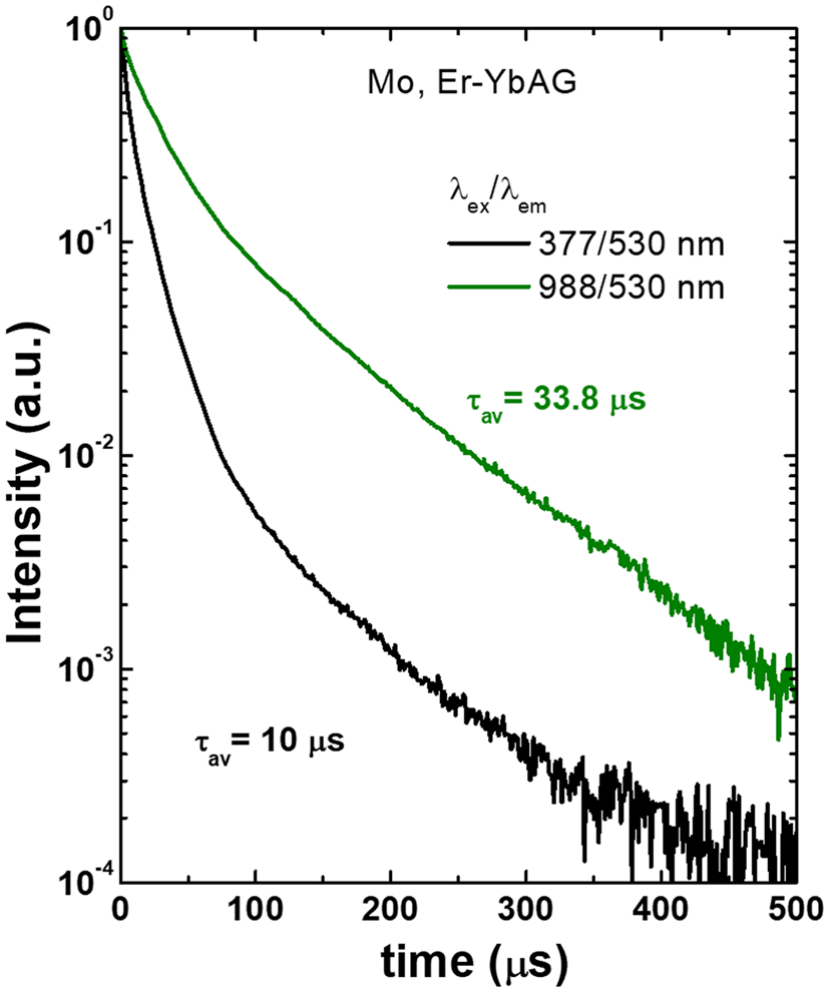
Er emission decays in Er, Mo -YbAG measured at 530 nm under direct (377 nm) and upconversion excitation (988 nm).

The double selective emission decays (in both excitation and monitored emission) further confirm the heterogeneous nature of the UC emission of Mo, Er-YbAG. In these measurements, we monitored the Er emission at 565 and 530 nm under selective excitation at 914 and 988 nm, respectively. As expected, the 565 nm decay is close to that measured in Mo-free YbAG, with an average decay time of 19 µs (Fig. S2). The 530 nm decay displays a longer transient with an average decay time of 34 µs (Fig. 5). More important is that the 530 nm decay is longer lived under upconversion (at 914 nm) than direct excitation (at 377 nm into Er ^4^I_15/2_-^4^G_9/2, 11/2_ absorption) with an average decay time of 10 µs. The result indicates that the Er emission in Mo, Er-YbAG occurs by a sequential Yb to Er energy transfer and not by GSA/ESA in the Mo-Yb dimer unit^35^.

**Figure 6:**
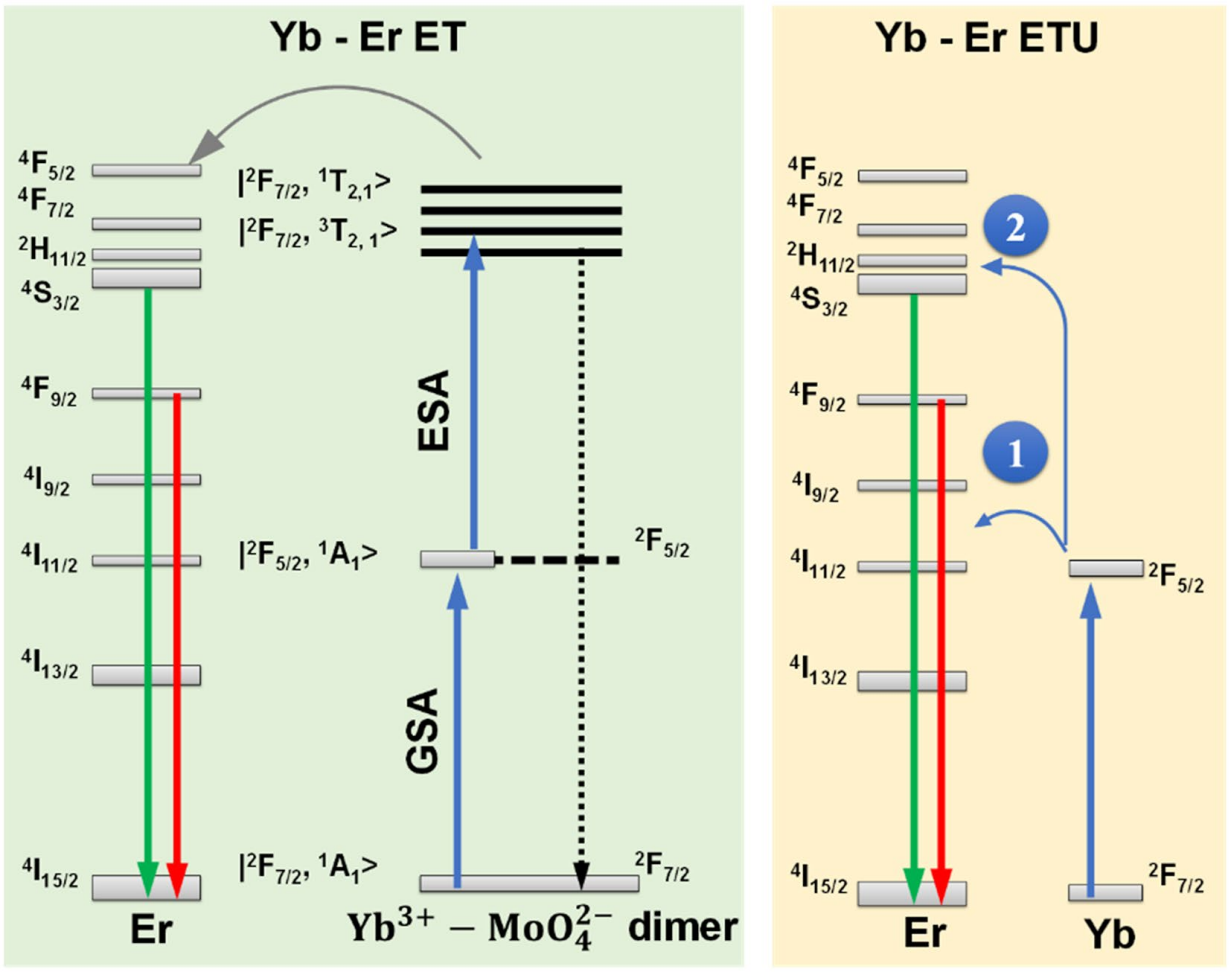
Upconversion mechanisms in the Mo-Yb dimer model *versus* standard sequential Yb-Er energy transfer (ETU). Numbered circles refer to Yb—Er ETU steps.

A comparison between the mechanisms occurring in a GSA/ESA Yb-Mo pair and ETU UC in TM (Mo) co-doped Yb, Er systems is illustrated in Fig. 6. In the former mechanism, the excitation into Yb absorption at 980 nm populates the ${\mathrm{Yb}}^{3+}-{\mathrm{MoO}}_{4}^{2-}$ state from the fundamental |^2^F_7/2_, ^1^A_1_ > to excited state |^2^F_7/2_, ^1, 3^T_2, 1_ > by a two-step sequence of GSA/ESA followed by direct energy transfer to Er ^4^F_5/2_ (at 485 nm)^9^. Direct energy transfer from the dimer excited state to Er ^4^F_5/2_ level with subsequent multiphonon relation on the (^2^H_11/2_, ^4^S_3/2_) leads to a selective enhancement of Er green (^2^H_11/2_, ^4^S_3/2_- ^4^I_15/2_) relative to red emission (^4^F_9/2_-^4^I_15/2_).

For ETU UC, Er ^4^I_11/2_ level is populated in step 1 via an energy transfer from Yb ^2^F_5/2_. Following a second energy transfer from Yb, the electron is promoted from ^4^I_11/2_ to ^4^F_7/2_ level. In Er-doped Yb garnets, red (Er ^4^F_9/2_-^4^I_15/2_) emission is favoured instead of green emission (^2^H_11/2_, ^4^S_3/2_- ^4^I_15/2_) through Er-Yb energy back transfer (Er ^4^F_7/2_-^4^I_11/2_ → Yb ^2^F_7/2_-^2^F_5/2_ and Er ^2^H_11/2_, ^4^S_3/2_-^4^I_11/2_ → Yb ^2^F_7/2_-^2^F_5/2_) and Er-Er cross relaxations (^4^F_7/2_-^4^F_9/2_ → ^4^I_11/2_ -^4^F_9/2_) that either fuel directly the ^4^F_9/2_ level or indirectly by increasing ^4^I_13/2_ level followed by subsequent ETU from Yb to Er ^4^I_13/2_ → ^4^F_9/2_^48^.

**Figure 7:**
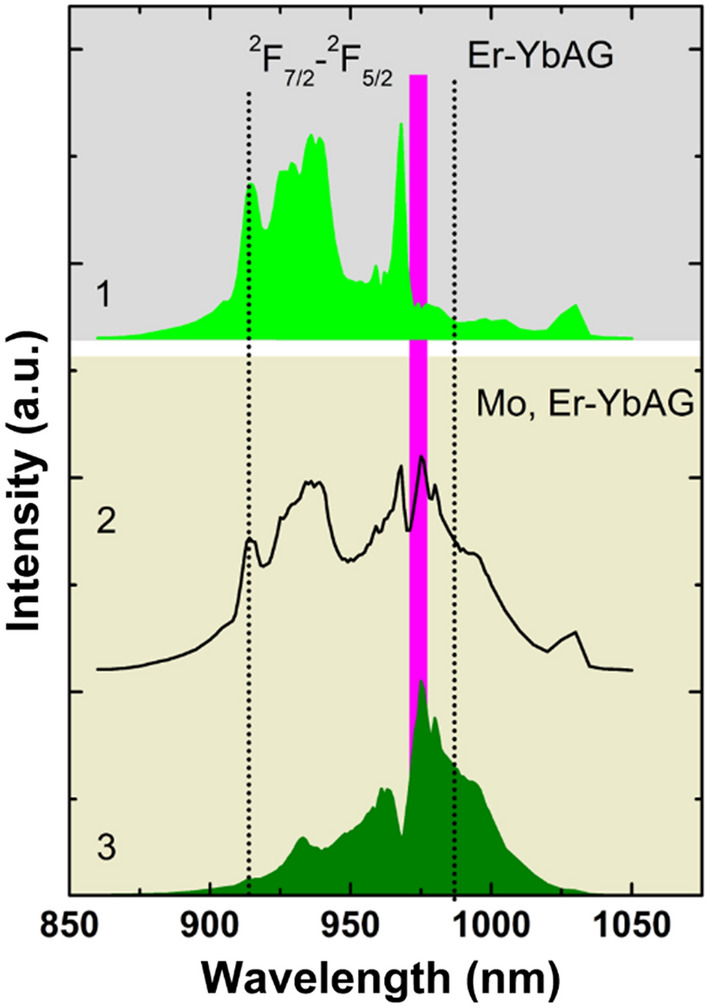
Upconversion excitation spectra monitoring Er green emission at 565 nm (Er-YbAG labelled as 1), 515–575 (Mo, Er-YbAG labelled as 2) and 530 nm (Mo, Er-YbAG labelled as 3); The magenta and black vertical lines correspond to the excitation wavelengths used in cw and pulsed laser excitation, respectively. The laser energy density is 20 mJ/cm^2^. All spectra are normalized at the maximum intensity.

Different to the absorption spectra, which measure global absorption, the excitation spectra reveal only the absorption that induces emission. Since Mo significantly modifies the Er upconversion emission, it is expected that the transition metal alters the Yb absorption as well. To this aim, we measured the UC excitation spectra of Mo free and Mo, Er-YbAG. In these experiments, the laser spanned the Yb absorption in the 875–1050 nm range with a 1 nm step, monitoring the Er green emission. Theoretical calculations^49^ have shown that, in the case of ETU mechanism, the UC excitation spectra of Er emission follow the Yb absorption narrowed by the power-law ${P}_{n}\left(\lambda \right)\approx {P}_{1}^{n}\left(\lambda \right)$, where n and λ refer to the emission order and the UC emission wavelength, respectively^35,49^. As illustrated in Fig. 7, without Mo, the excitation spectrum of Er green emission (565 nm, spectrum 1) replicates in shape the Yb squared linear absorption spectrum illustrated in Fig. 1c. This unsurprisingly confirms that the Er green emission is Yb sensitized by the sequential ETU two-photon process.

It is well-known known that the shape of the UC excitation spectra depends on the mechanism involved, ETU or GSA/ESA^35^. Since Mo has no absorption around 980 nm, the |^2^F_7/2_, ^1, 3^T_2, 1_ > excited state (Fig. 6) being purely virtual, the excitation spectrum of Mo, Er-YbAG should follow the Yb absorption, irrespective of the UC mechanism, GSA/ESA (dimer sensitization) or classical ETU. This is not the case, as the excitation spectra indicate that Mo induces an additional Yb absorption. Although this additional Yb absorption contributes by more than 40% to the total excitation UC spectra (estimated by spectral deconvolution in Fig. 7), it falls below the detection limit of the standard optical absorption technique (Fig. 1c). Besides the ability to reveal absorptions not detectable by optical measurement, the excitation UC spectra determine the value of the excitation wavelength needed for an accurate comparison between samples. Cw excitation at 973 nm used here or between 975 and 980 nm reported in literature^9,47^ fall in a low Yb absorption valley in Mo-free Er-YbAG while they match the maximum Yb absorption in Mo, Er-YbAG is leading to an incorrect comparison of the emission intensity.

For Mo, Er-YbAG, we measured the excitation spectra monitoring the Er green emission in both global/non-selective and selective modes, monitoring the whole emission between 515 and 570 nm or within a narrow range of ± 2 nm around 530 nm, respectively. It is apparent that the non-selective excitation spectrum (spectrum 2) is broader and more complex than the spectrum of Mo-free YbAG while including the spectral features of the excitation spectrum measured for Mo-free YbAG. The selective excitation spectrum (spectrum 3) displays a significantly altered shape with the peak maximum shifted by 7 nm, from 968 (Er-YbAG) to 975 nm (Mo, Er-YbAG). It is this Yb absorption that sensitizes the Er green UC emission in Mo, Er -YbAG. Spectrum 2 can be readily constructed as a linear convolution of spectra 1 and 3 which confirms the co-existence of two Yb absorptions in Mo, Er-YbAG. The co-existence of two Yb absorptions in Mo, Er-YbAG explains the dependency of Er UC emission spectra on the excitation wavelength (Fig. 3).

### Local structure investigation

We further investigated whether Mo alters the local structure around Er by using Eu as a luminescence probe^49^. To this aim, we synthesized two samples of (Mo), Eu-YbAG (XRD patterns are shown in Fig. S3). As illustrated and discussed in Fig. S4, Mo significantly alters the Eu emission and excitation spectra relative to those measured with Mo-free Eu-doped garnet^50^.

In YbAG Eu substitutes for Yb in the D_2d_ site symmetry give rise to relatively strong magnetic dipole ^5^D_0_-^7^F_1_ triplet lines followed by weaker electric dipole ^5^D_0_-^7^F_2_ emission with peaked around 610 nm in perfect agreement with the literature^50^. Adding Mo significantly changes the emission and excitation properties (Fig. S4). The emission is dominated by the ^5^D_0_-^7^F_2_ transition presenting a distinctive doublet splitting at 613 and 616 nm, while the excitation spectrum displays a broad UV absorption around 305–310 nm which is absent in the Mo-free sample. Both emission and excitation spectral shapes (such as the broad UV absorption of the O^2−^Mo^6+^ group) are highly similar to those reported for Eu-RE_2_(MoO4)_3_^51–53^. We further checked this by spectroscopic investigation of our sample of Eu-Yb_2_(MoO_4_)_3_ sample (XRD patterns shown in Fig. S3).

In Eu- Yb_2_(MoO_4_)_3_, upon substitution of Yb in the low symmetry monoclinic sites (*C*_2_/c)^54^ Eu displays a relatively strong ^5^D_0_-^7^F_2_ emission with doublet lines at 613 and 616 nm, which superimpose perfectly on the emission of Mo, Eu- YbAG.

**Figure 8:**
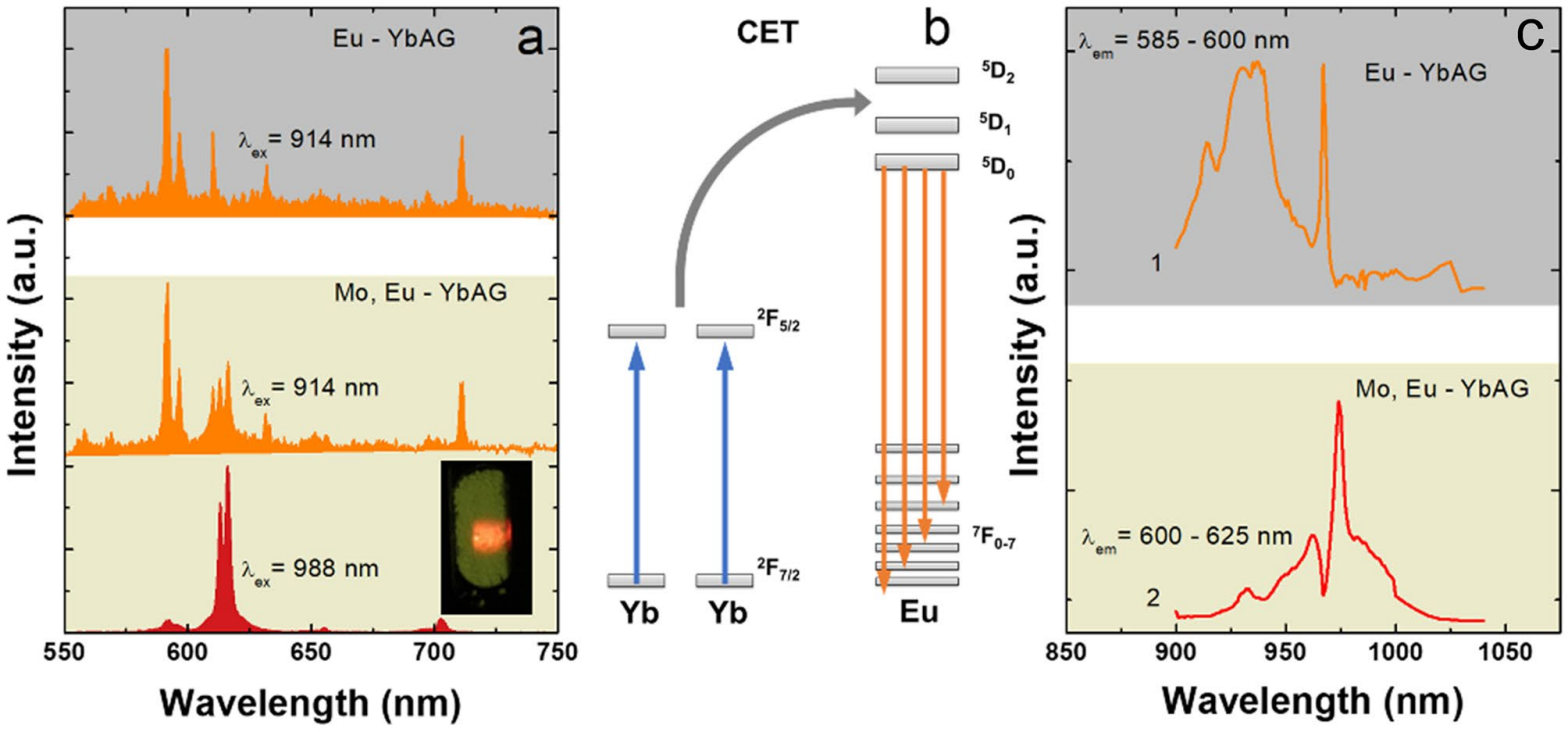
Eu upconversion emission (**a**) and excitation spectra (**c**) of (Mo), Eu-YbAG; A schematic illustration of the Yb-Eu cooperative upconversion (CET) mechanism is also included (**b**). The digital photo presents the Mo, Eu-YbAG emission upon 988 nm excitation.

Local structure investigations suggest that Er is surrounded by a similar oxygen environment in the Mo co-doped YbAG and Yb_2_(MoO_4_)_3_. To correlate further the local structure and upconversion emission, we used variable excitation across Yb absorption and measured the Eu emission. A relatively weak Eu UC emission in Mo-free YbAG is detected, which is greatly intensified in the Mo co-doped YbAG (Fig. 8a). The Eu UC emission in Mo, Eu-YbAG is a linear superposition of two spectra (Fig. S5), characteristic of Eu emission in garnet and Yb_2_(MoO_4_)_3_.

The spectra are best discriminated using excitation at 914 and 988 nm, respectively, similar to Mo, Er-YbAG. It is recognized that the UC emission in Yb to Eu is orders of magnitude less efficient than in Yb, Er couples^35^. In most cases, the mechanism is of cooperative nature (CET), although, in specific Mn, Eu, Yb systems, it is assigned to the Yb-Mn(2 +) dimer sensitization^16,22–24,55^. In the cooperative UC (Fig. 8b), two Yb sensitizers in the excited state transfer energy to the ^5^D_2_ level of Eu, which relaxes non-radiatively on the ^5^D_0_ emitting level^56^. We further measured the excitation spectra (Fig. 8c), monitoring the UC emission of Eu in Mo-free and Mo co-doped YbAG under laser scanning the Yb absorption from 900 to 1050 nm. In these experiments, the Eu emission was monitored from 585 to 600 nm (Eu-YbAG) and 600 to 625 nm (Mo, Eu-YbAG), respectively. As expected, the UC excitation spectrum of Eu emission in Mo-free YbAG matches the Yb absorption in YbAG as Eu emission is Yb sensitized. The notable result is that the UC excitation spectrum of Eu emission in Mo, Eu-YbAG (Fig. 8c) matches the Yb absorption measured in Mo, Er-YbAG (Fig. 7). Collectively, the Eu data narrow the possible structures for the Mo-induced impurity phase in YbAG to RE_2_(MoO_4_)_3_.

The lower local symmetry at Ln sites in the Mo oxide compared to YbAG (C_2_ versus D_2d_)^57^, along with a reduced Er/Yb concentration which reduces the efficiency of deleterious cross-relaxations, and thus the amount of luminescence quenching^58^ contributes to the higher intensity of Er UC emission in Mo co-doped YbAG relative to Mo free counterpart.

### TEM investigations on multiple grains

**Figure 9:**
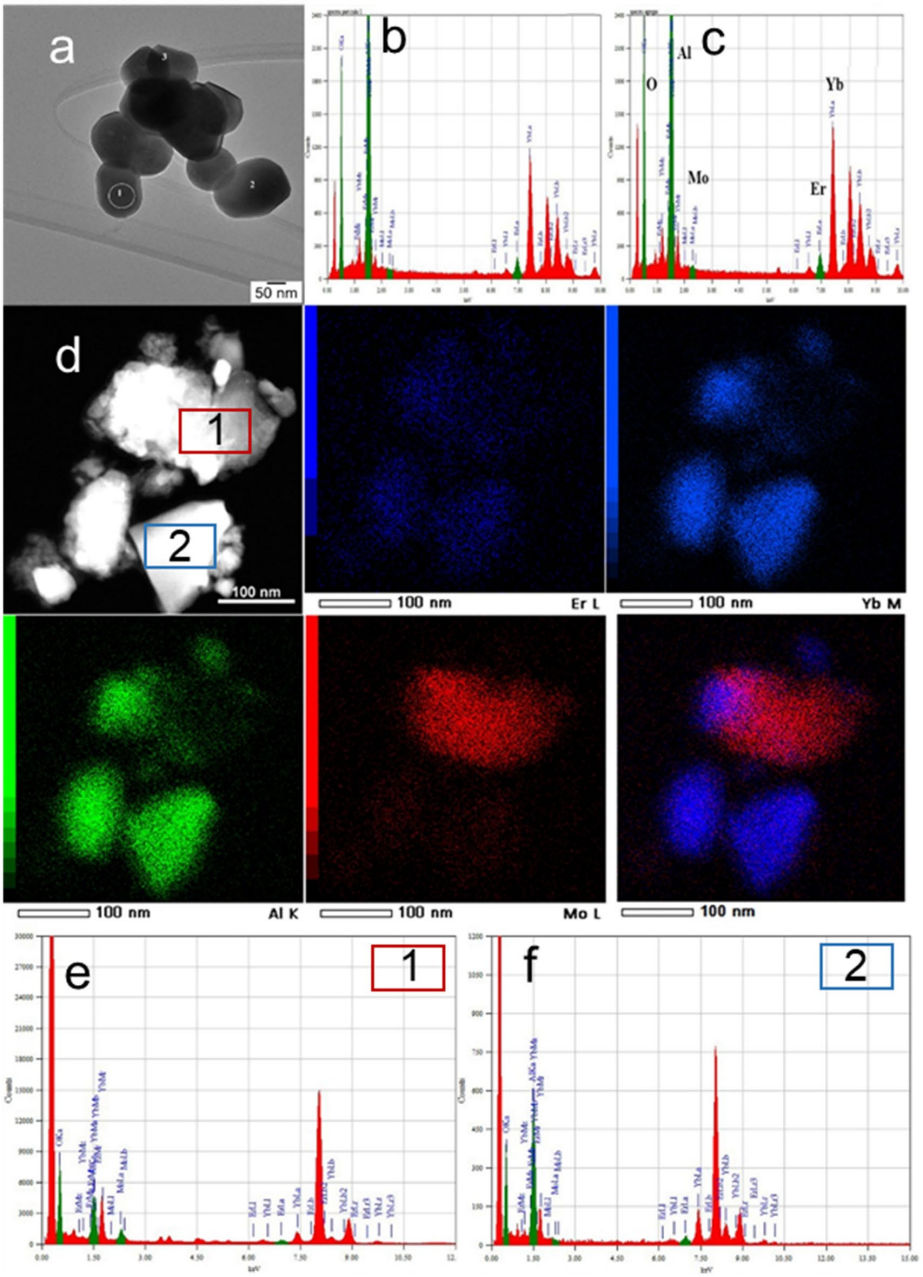
(**a**) Low magnification TEM image of an aggregate of Mo, Er-YbAG crystallites (**b,c**) EDX spectra measured on the crystallites indicated by (1) and (2) on the aggregate in (**a**); (**d**) HAADF-STEM image and the corresponding elemental mapping of Mo, Yb, Al and Er elements on a Mo, Er-YbAG aggregate; (**e,f**) EDX spectra acquired from the areas delimited by the rectangles 1 and 2 showing Mo depleted (1) and Mo enriched particles (2) the latter being enclosed by the Mo free YbAG particles. The Er doping level was subjected to gross errors due to overlapping between the M-lines of Yb, Er elements and L-line of Al element.

We further used high-resolution transmission electron microscopy (TEM) on multiple grains to check whether the TM and Ln metals distribute uniformly on the nanoscale range. We note that the investigation was pursued on tens of grains and spanned several months. According to TEM images (Fig. 9), the crystallites of (Mo)Er-YbAG are agglomerated, with dimensions between 50 and 200 nm, and monocrystalline with some facets.

The crystallite morphology is similar in Mo-free, and Mo co-doped YbAG, with a slightly smaller crystallite size, observed for the latter sample. Al_2_O_3_ or alumina inclusion with a contribution of up to 7.8% has been detected by XRD in all garnet samples investigated (Mo), Er/Eu-YbAG. Alumina formation is typically observed in garnets being associated with a Y/Yb surplus^39^.

Figure 9a shows a typical aggregate of small crystallites of Mo, Er-YbAG. EDX spectra in Fig. 9b,c indicate that the level of Mo doping is low (0 to 0.9%) and slightly varying across crystallites. The analysis of a second aggregate by STEM-EDS mapping in Fig. 9d highlights that the Mo and Yb elements distribute non-uniformly across the Mo-doped Er-YbAG particles. EDX mapping on crystallite 1 in Fig. 9e shows again depleted Mo particles with a Mo level doping close to 0%. However, rare Mo-enriched particles with Mo-level doping as high as 90% are also detected (crystallite 2 in Fig. 9f), which are enclosed by Mo-free YbAG crystallites. Although we could not identify by TEM the crystalline structure of the Mo-rich particles, we can associate these with the formation of Er, Yb, Mo oxide, close to Yb_2_(MoO_4_)_3_ as identified by the multistep spectroscopic approach described above.

Overall, TEM analysis of Mo, Er-YbAG shows a co-existence of Mo depleted (Er-YbAG) and Mo rich particles (Er-Yb_2_(MoO_4_)_3,_ which determines the heterogeneity effects revealed by spectroscopic investigations.

## Conclusions

We investigate whether the effects induced by a TM co-dopant (i.e. Mo) on the Er UC emission are ascribed to Mo-Yb dimer sensitization as currently accepted in the literature. To this aim, we employ a pulsed and tunable excitation laser under controlled energy density. Unlike fixed, steady-state excitation employed so far, pulse excitation allows the clarification of the upconversion mechanism and the presence of multisite, dopant or phase segregation. Collectively, our results sustain that the Er UC emission behaviour induced by Mo is associated with a chemical impurity with a molybdate lattice structure. In this Mo phase, a relatively efficient upconversion occurs via Yb to Er sequential energy transfer. Since of all hosts investigated so far, the garnet structure offers the greatest tolerance towards co-doping with transition and lanthanide metals. The results may be illustrative for other systems to predict that dimer sensitization is an implausible process.

## Supplementary Information

Supplementary Figures.

## Data Availability

The datasets used and/or analysed during the current study are available from the corresponding author on reasonable request.
