# Supplementary material for: Up-conversion emission in transition metal and lanthanide co-doped systems: dimer sensitization revisited
Source: Sci Rep. 2023 Feb 7;13:2165. doi: 10.1038/s41598-023-28583-3 (PMC9905471; doi:10.1038/s41598-023-28583-3)
Supplement: Supplementary file 1 — Supplementary Figures. [file 41598_2023_28583_MOESM1_ESM.pdf]

Supporting Information

Up-conversion emission in transition metal and lanthanide co-doped systems: dimer sensitization revisited

Daniel Avram[1], Claudiu Colbea[2], Andrei A. Patrascu[1], Marian Cosmin Istrate[3, 4], Valentin Teodorescu[3, 5] and Carmen Tiseanu[*, 1]

[1] *National Institute for Laser, Plasma and Radiation Physics, PO Box MG-36, RO 76900 Bucharest-Magurele, Romania*

[2] *Scientific Center for Optical and Electron Microscopy, ETH Zürich, Zürich, Switzerland*

[3] *National Institute of Materials Physics, 405A Atomistilor Street, 077125 Magurele-Ilfov, Romania*

[4] *University of Bucharest, Faculty of Physics, 077125 Magurele, Romania*

[5] *Academy of Romanian Scientists, 050094 Bucharest, Romania*

[*] *E-mail: carmen.tiseanu@inflpr.ro*

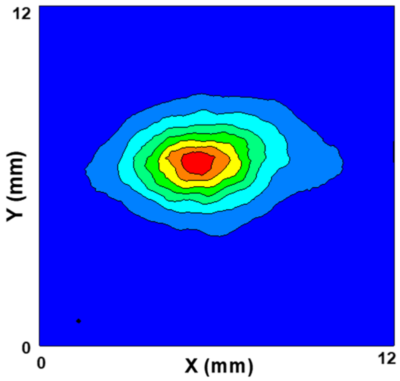

Figure S1. Gaussian beam profile of OPO laser used in the emission spectra, decay and excitation spectra measurements.

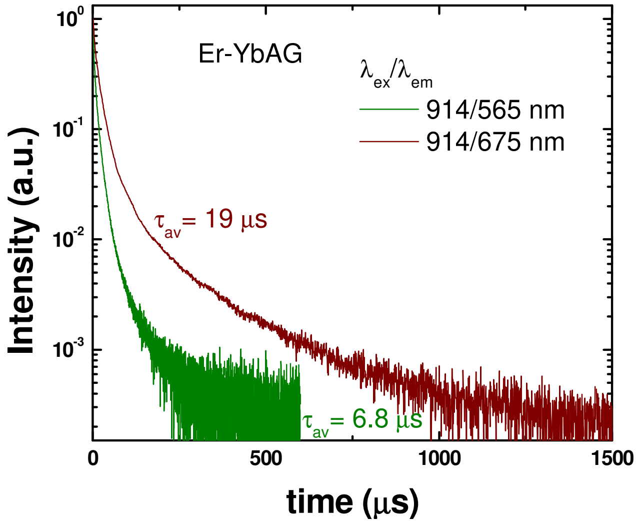

Figure S2. UC emission decays of Er-YbAG monitoring Er emission at 565 and 675 nm under 20 mJ/cm^2 excitation at 914 nm.

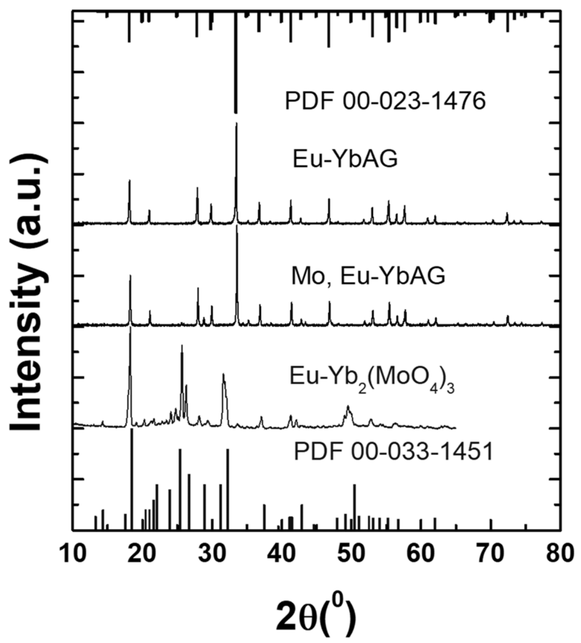

Figure S3. XRD patterns of (Mo), Eu YbAG and Eu-$Yb_2(MoO_4)_3$.

Shown in **Figure S3** are the XRD patterns of Eu-YbAG and Mo, Eu-YbAG consistent with the YbAG structure (PDF card 00-023-1476) with a small content of ~4% Al_2O_3 phase (calculated using MAUD software). The XRD pattern of Eu-$Yb_2(MoO_4)_3$ is described by PDF card 00-033-1451, corresponding to $Yb_2(MoO_4)_3$ structure, space group *C*2/c. The crystallite sizes estimated by Scherrer equation[5] are 76, 52 and 25 nm for Eu-YbAG, Mo, Eu-YbAG and Eu-$Yb_2(MoO_4)_3$.

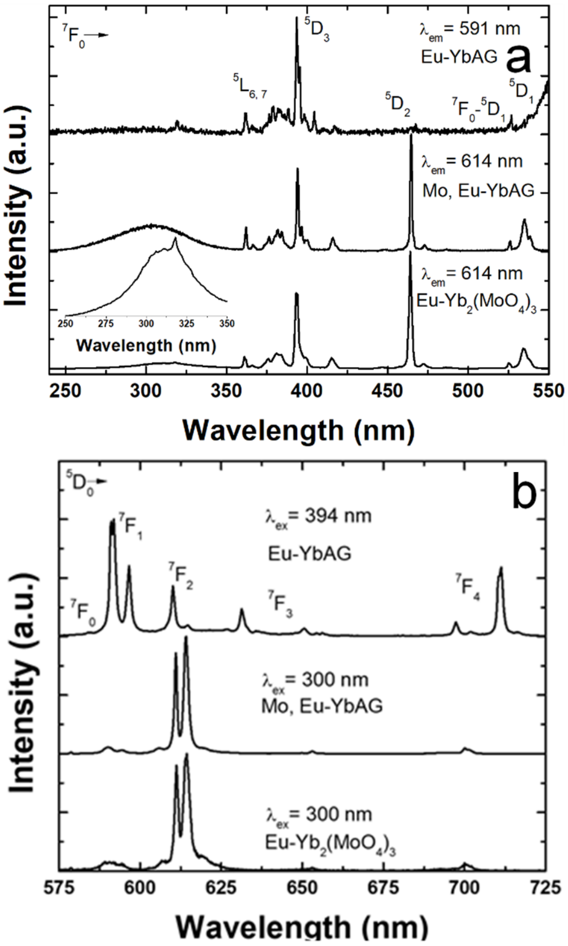

Figure S4. Eu emission and excitation spectra in (Mo), Eu-YbAG and Eu-$Yb_2(MoO_4)_3$.

In YbAG Eu substitutes for Yb in the D_{2d} site symmetry giving rise to relatively strong magnetic dipole $^5D_0$ - $^7F_1$ triplet lines followed by weaker electric dipole $^5D_0$ - $^7F_2$ emission with peaked around 610 nm in perfect agreement with the literature[6]. The addition of Mo significantly changes the emission and excitation properties **(Figure S4a, b)**. The emission is dominated by the $^5D_0$ - $^7F_2$ transition presenting a distinctive doublet splitting at 613 and 616 nm, while the excitation spectrum displays a broad UV absorption around 305-310 nm which is absent in the Mo-free sample. Both emission and excitation spectral shapes (such as the broad UV absorption of the O^{2-}-Mo^{6+} group) are highly similar to those reported for Eu- $RE_2(MoO4)_3$ [7-9]. To further confirm this, we synthesize and characterize Eu- $Yb_2(MoO4)_3$. In this host, upon

substitution of Yb in the low symmetry monoclinic sites (*C*2/c) [10] Eu displays a relatively strong $^5D_0$ - $^7F_2$ emission with doublet lines at 613 and 616 nm which superimposes perfectly on that of Eu in YbAG [11].

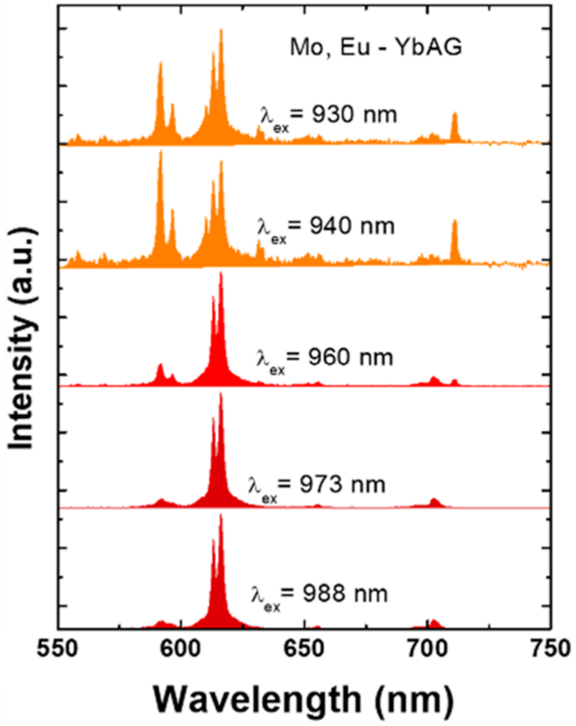

Figure S5. Upconversion emission spectra of Mo, Eu-YbAG using variable pulse excitation (at 20 mJ/cm^2) across Yb absorption (930 – 988 nm).

References

1 Dong, B. *et al.* Temperature Sensing and In Vivo Imaging by Molybdenum Sensitized Visible Upconversion Luminescence of Rare-Earth Oxides. *Advanced Materials* **24**, 1987-1993 (2012). https://doi.org:10.1002/adma.201200431

2 Tian, X. *et al.* Investigation of anomalous upconversion luminescence in YbAG: Er, Mo. *Materials Letters* **123**, 48-50 (2014). https://doi.org:10.1016/j.matlet.2014.02.076

3 Tiseanu, C. *et al.* Structural, down- and phase selective up-conversion emission properties of mixed valent Pr doped into oxides with tetravalent cations. *Physical Chemistry Chemical Physics* **16**, 5793-5802 (2014). https://doi.org:10.1039/c3cp54899f

4 Avram, D. *et al.* Imaging dopant distribution across complete phase transformation by TEM and upconversion emission. *Nanoscale* **11**, 16743-16754 (2019). https://doi.org:10.1039/c9nr04345d

5 Patterson, A. L. The Scherrer Formula for X-Ray Particle Size Determination. *Physical Review* **56**, 978-982 (1939). https://doi.org:10.1103/PhysRev.56.978

6 Pavasaryte, L. *et al.* Eu3+- Doped Ln(3)Al(5)O(12) (Ln = Er, Tm, Yb, Lu) garnets: Synthesis, characterization and investigation of structural and luminescence properties. *Journal of Luminescence* **212**, 14-22 (2019). https://doi.org:10.1016/j.jlumin.2019.04.005

7 Xu, Z. *et al.* Self-Assembled 3D Urchin-Like NaY(MoO4)(2):EU3+/Tb3+ Microarchitectures: Hydrothermal Synthesis and Tunable Emission Colors. *Journal of Physical Chemistry C* **114**, 2573-2582 (2010). https://doi.org:10.1021/jp9115029

8 Zhou, Y. & Yan, B. RE2(MO4)(3):Ln(3+) (RE = Y, La, Gd, Lu; M = W, Mo; Ln = Eu, Sm, Dy) microcrystals: controlled synthesis, microstructure and tunable luminescence. *Crystengcomm* **15**, 5694-5702 (2013). https://doi.org:10.1039/c3ce40495a

9 Wang, S. *et al.* Structural Characterization and Luminescent Properties of a Red Phosphor Series: Y2-xEux(MoO4)(3) (x=0.4-2.0). *Journal of the American Ceramic Society* **92**, 1732-1738 (2009). https://doi.org:10.1111/j.1551-2916.2009.03118.x

10 Atuchin, V. *et al.* Synthesis and Spectroscopic Properties of Monoclinic alpha-Eu-2(MoO4)(3). *Journal of Physical Chemistry C* **118**, 15404-15411 (2014). https://doi.org:10.1021/jp5040739

11 Kaczmarek, A. & Van Deun, R. Rare earth tungstate and molybdate compounds - from 0D to 3D architectures. *Chemical Society Reviews* **42**, 8835-8848 (2013). https://doi.org:10.1039/c3cs60166h
